# Is self-testing the next paradigm for diagnostics?

**DOI:** 10.6026/97320630019278

**Published:** 2023-03-31

**Authors:** Diptaraj S. Chaudhari, Vikas Ghattargi, Viqar Shah, Saurabh Gupta, Kedar Bele, Shrikant Pawar

**Affiliations:** 1 Mylab Discovery Solutions Private Limited, Pune, India; 2 Netsurf Communications Pvt. Ltd., Pune, India

**Keywords:** Usability assessment, AI powered Mylab Coviself™ application, age and gender, Ease of testing process, Mylab CoviSelf™ - COVID-19 Rapid Antigen Self-Test Kit

## Abstract

The study estimates the usability and attitude assessment of users for India's first approved rapid antigen self-test kit; the CoviSelf™. India approved its first AI-powered self-test for Covid-19 in April 2021 a few weeks after the first approval in the US. We present here a study on usability and attitude assessment of users of India's first approved rapid antigen self-test kit; the CoviSelf™. The study evaluates participants' understanding of and performance of test procedure and interprets the results. Analysis revealed that more than 90% study participants followed steps correctly as illustrated in the user's manual. Age group and gender-based analysis showed comparable scores for usability of the test kit suggesting users of different age groups has same ease in using the test kit. What we learnt from this study could be start of self-test revolution, where rapid tests could expand the access of diagnostics for hundreds of diseases including HIV, HPV, and dengue to millions of people who could not get access to diagnostics because we lacked manpower or facility to conduct tests. Self-testing could break the barriers for diagnostics that Internet did for information.

## Background:

The first case of severe acute respiratory syndrome coronavirus 2 (SARS-CoV-2) known as COVID-19 was reported in China in December [1,2]. By March 2022, more than 665 million people worldwide were infected and more than 6 million lost their lives. India reported more than 44 million infections of COVID-19 by January 3, 2022 (worldometers.info). This pandemic challenged public health systems worldwide in their effort to save human lives [3]. Frequent lockdowns left many at risk of sinking into poverty [4]. SARS-CoV-2 transmission occurs through air contaminated by droplets and airborne particles. The infected show fewer, cough, headache as most prevalent symptoms [5,6], however, of the infected only ~73% infected population develops symptoms and every one in four (~27%) remains asymptomatic, who can transmit virus unknowingly and pose challenge to curb disease transmission [7]. In addition to preventive methods prescribed by world health organization (WHO) to control the spread, early detection and isolation of the infected plays a key role in slowing down transmission [8]. For detection, three broad diagnostic techniques are prevalent: molecular testing Reverse transcriptase polymerase chain reaction (RT-PCR), Next-generation sequencing (NGS based, antigen rapid testing and antibody rapid testing [8,9,10]. In India, with just 2 labs equipped to test for Covid-19 at start of pandemic in January 2020, molecular testing infrastructure was almost non-existent and was built as the pandemic progressed. By September 2021, India had ~1500 certified molecular labs, one for every ~ 901761 people, and largely concentrated in urban centers (https://nabl-india.org/). During the second wave of pandemic, India experienced severe shortage of testing infrastructure. Average turnaround time of sample processing increased to more than 72 hours even in metro cities with best lab infrastructure. To address this gap, ICMR decided to shift to rapid antigen test (RAT) as its primary testing technique because the molecular lab infrastructure was not sufficient, positivity rates were high and RAT could provide results within 30 minutes [11,12]. However, traditional rapid antigen test (RAT) needed to be performed by a healthcare worker in a healthcare setting. This presented a huge logistic challenge because of shortage of, and overworked healthcare workers [13]. This led Indian council of medical research (ICMR) to consider and approve home use self-detection kit for COVID 19 infections based on rapid antigen test. Mylab's CoviSelf™ was the first ICMR-approved self-testing kit for Covid-19 in India. CoviSelf™ was also the first ever self-test for disease diagnosis in India. ICMR carefully considered the usability aspects so that test could be used despite social, economic, and linguistic diversity in India. The testing process needed to be safe and simple to follow, be provided with visual aids, and give automated interpretation (ICMR Advisory for COVID-19 Home Testing using Rapid Antigen Tests (RATs) Dated 19.05.2021). The design of self-test solution was closely monitored by ICMR and a test was finally approved on May 20th 2021 in India. It is estimated that more than 10 million self-tests have been ordered in India since their launch (as per conversations with executives of companies). In this paper, we study patients' ability to safely and correctly use a self-test and explore if self-testing could become the solution for containment of many other diseases in the developing world.

## Methods:

We used the Mylab CoviSelf™ COVID-19 Rapid Antigen Self-Test Kit developed by Mylab Discovery Solutions Pvt Ltd of India for the usability assessment. We conducted a usability testing study to understand how subjects used the India's first ICMR approved Covid-19 Rapid antigen self-test kit. The study was performed with 150 study participants across socio-economic status and education level. The study participant involved in this survey belonged to different socioeconomic status ranging from daily wage workers (n=99), office staff (n=28), food industry staff (n=18), maids (n=6), household members (n=12) involving both male (n=55) and female participants (n=95). The age of the participants was ranging from 15 to 50 years. The consent of the participants was also taken for their participation in the study. This study will serve as a reference in understanding if users can perform self-tests for disease diagnosis effectively and to understand the user attitude towards self-testing.

The Mylab CoviSelf™ - COVID-19 rapid antigen self-test kit is based on lateral flow principle and has five components including sterile nasal swab, prefilled lysis buffer tube, QR-coded test card, biohazard bag, and instruction for use (IFU) manual. The user or someone on their behalf can use the CoviSelf™ mobile App to view instruction video in local language, interpret test result and to report it to ICMR server. Each testcards are confined with the unique quick response (QR) code for assignment of report number generated directly from the Indian council of Medical Research (ICMR) portal. The details for the overall test procedure have been explained in user manual for the self-test.

The CoviSelf™ app (available on Google Play Store & Apple App Store in India) is a uniquely designed app based on the artificial intelligence (AI) model wherein, the image or picture of the testcard needs to be scanned and the model predicts the result. We developed an image processing model using Convolutional Neural Networks (CNN) which was trained on 100,000 images which were generated synthetically from 300 real images with different concentrations of attenuated viral antigen samples. This was an important step as the app were to be used by different people in different conditions - with variation in lighting, background, presence of absence of shadow, orientation of the cassette. These images helped develop a very accurate model which could easily create bounding boxes and predict results with 98.6% accuracy.

## Design of questionnaires and observation tables:

Our usability testing framework was informed by

1) Guidance document - applying human factors and usability engineering to medical devices guidance for industry and food and drug administration staff of February 2016

2) EN 62366-1:2015 Medical devices - Part 1: Application of usability engineering to medical devices

3) ISO 14971:2019 Medical devices - Application of risk management to medical devices guidelines for usability

The usability was evaluated on parameters which can be categorized into three fundamental dimensions including (1) Ability to understand/follow Instructions to test, (2) Performance of test procedure and (3) Interpretation of test result. Questions relating to use's safety, effectiveness of the test kit and procedure, ease of sample collection, testing process, result interpretation, understating the user manual and labels were asked in the questionnaire.

## Data collection and analysis:

The study participants were provided with the Mylab CoviSelf™ COVID-19 rapid antigen self-test kit and the user feedback form. The users were instructed/demonstrated for use of device or were asked to read the user manual provided in the kit. The user was monitored by trained moderators for the use of provided kit. The observations and errors were recorded by the moderators at each step. After the successful self-testing the user were asked to fill the user feedback related to their understating of user manual and labels, ease of sample collection, testing process, result reporting. Also, they recorded their concerns on safety and overall satisfaction with the test kit.

## Data Analysis:

Responses recorded by the study participants after using the Mylab CoviSelf™ COVID-19 rapid antigen self-test kit were tabulated into the data matrix. Also, the observations noted by the trained moderators appended to the data matrix for each participant. These values were then aggregated and transformed into the percent values for each study parameter. User's feedback was also transformed into the percent values. Analysis of variance (ANOVA) was performed for the comparison of satisfaction scores recorded by the participants across socioeconomic status and age groups and t-test based analysis was performed between the members of gender using GraphPad Prism (Chaudhari et al., 2020). We also separately evaluated responses of participants from different age groups and gender, wherein for the age group-based analysis study participant were grouped into four groups from 18 years up to 50 years of age.

## Results:

Data matrix generated based on the observations of the trained moderators was used for the analysis. Analysis revealed that more than 90% study participants followed all the steps correctly as illustrated in the user's manual and demonstrated by the technician. 93% users handled the sterile swab carefully (n=140) and effectively collected the sample. 94% (n=141) users dipped the swab into the buffer after taking sample. 93% users handled the test cassettes properly and 92% followed the proposal protocol for disposed of test cartridges. Also, 99% of the study participants observed the cassette for 15 mins for final conclusion of the test results (Table 1). It was noted that ~17% study participants had final extract with small amount of mucus, however, it did not hamper the flow of the buffer and the test ran successfully.

## User feedback: overall satisfaction with the kit procedure:

**Figure 1:**
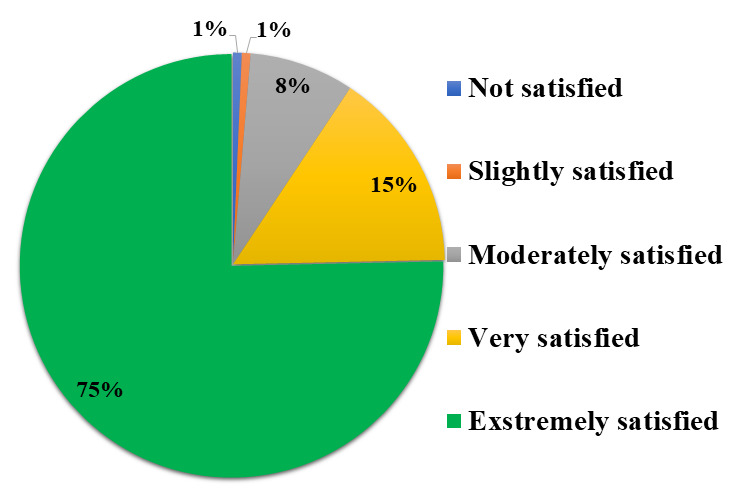
Pie chart depicting the overall satisfaction rating of the user to Mylab CoviSelf™ - COVID-19 rapid antigen self-test kit.

Users were asked to offer their scores to different satisfaction parameters ranging from 1 (not satisfied) to 5 (extremely satisfied). The overall satisfaction score of the users was 4.4 out of 5. Overall, it has been noted that 75% study participants were extremely (Score 5), 15% were very satisfied (Score 4), 8% were moderately satisfied (Score 3) while only 1% were slightly satisfied (Score 2) and 1% were not satisfied (Score 1) with the self-testing process using Mylab CoviSelf™ - COVID-19 rapid antigen self-test kit (Figure 1). We have noted that, amongst the different survey parameters, the lowest value (i.e., 1) was opted by 4% patient to the parameter, ease of understating the user manual and labels while ease of testing process was highly rated (score 5) parameter opted by ~78% study participants (Table 3).

## User feedback: based on user's socioeconomic status:

Here, the study participants have different job profile to represent various socioeconomic strata of population. Analysis of results suggested that in all these groups the usability ratings were comparable across these groups suggesting user-friendly applicability irrespective of the socioeconomic status (Table 2).

## User feedback: based on age group and gender:

**Figure 2:**
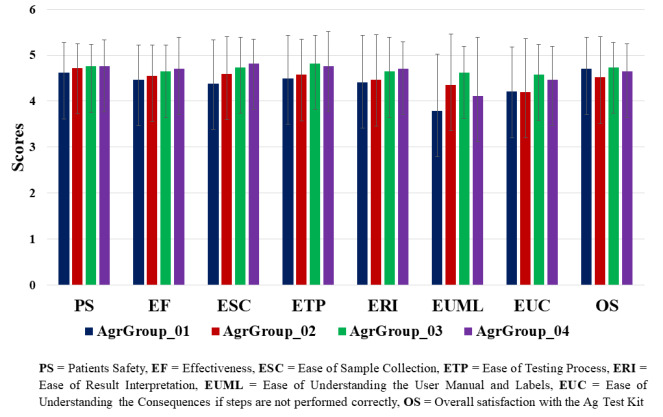
Bar plot representing the overall satisfaction rating of the user from different age group to Mylab CoviSelf™ - COVID-19 rapid antigen self-test kit.

**Figure 3:**
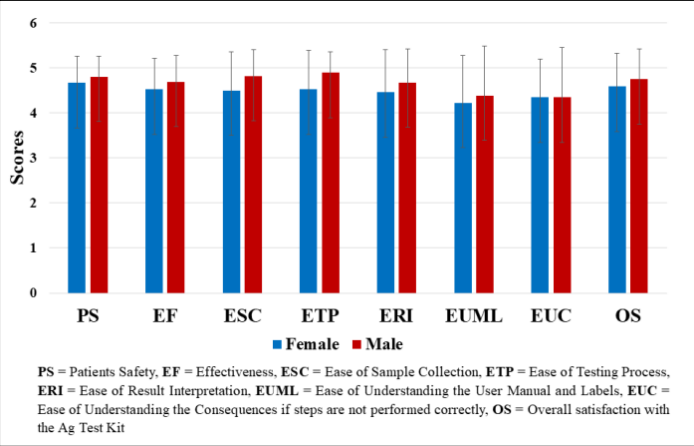
Bar plot representing the overall satisfaction rating based on user's gender to Mylab CoviSelf™ - COVID-19 rapid antigen self-test kit.

We have analyzed user responses to the overall usability of the test kit by age groups. We grouped the users into four groups wherein, first age group included participants from 18 to 20 years, second age group between 21 to 30 Years, third age group between 31 to 40 years and fourth age group between 41 to 50 years. Here, we have observed that in the first, second, third and fourth age groups the number of participants were 34, 54, 45 and 17, respectively. The average overall satisfaction rating with the Ag test kit was ≥4.5 across all the age groups. Age group-based analysis also showed comparable scores for the usability of the test kit suggesting users of different age groups have same ease in using the test kit (Figure 2). Similarly, we have performed the analysis based on the gender of the participants and we have recorded that overall, the effect was comparable across the gender groups (Figure 3).

## Discussion:

For the detection of Covid-19 molecular and rapid antigen detection measures were used and RAT were extensively used by trained manpower in laboratory settings for rapid screening of the massive population [14, 15]. Considering the testing onus on the manpower, ICMR has presented the advisory for COVID-19 Home Testing using RAT [16]. Here, in the present study we have presented the usability evaluation of ICMR approved, first covid test kit for home use i.e., CoviSelf™ [16]. We have examined the precision of users in self-testing and interpretation of the test results suggested in the IFU of the CoviSelf™. For the unbiased comparison the participant were selected from different age groups, gender and socioeconomic status. We have noted that the study participants from distinct groups (Figure 1,Table 2) have performed the test procedures correctly. It has been observed that comparable response in terms of ease of understanding instructions, following test process and satisfaction were offered by the users while self-testing suggested that this modality of home test can be very effectively used by the common public and does not demand any specific training (Figure 1,Table 2). Self-testing using CoviSelf™ was correctly done by more than 90% study participants who have followed all the steps correctly as illustrated in the user manual and demonstrated by the technician (Table 1). This signifies solemnness of study participants in precise understanding of the COVID-19 infection state because human nature is to take short cuts, if things are complicated and efforts demanding.

**Figure 4:**
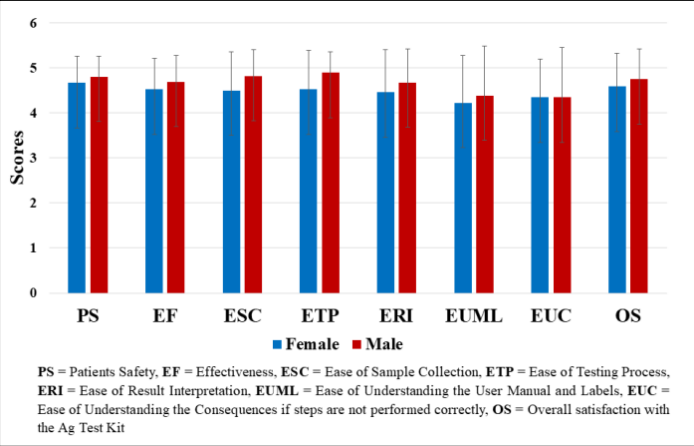
Prototype model

We have noted that, amongst the different survey parameters, the lowest value (i.e., 1) was opted by 4% patient primarily due to linguistic issues (Table 1). Language barrier perceived to be the responsible factor, as earlier the user manual was only provided in the one (English) language only. After suggestions by the user in the feedback form, we have included other regional languages in the IFU. Age group and gender-based analysis also showed comparable scores for the usability of the test kit suggesting users of different age groups has same ease in using the test kit (Figure 2, Figure 3, Figure 4). The Mylab CoviSelf™ application collects and uploads the demographic information of the users to the ICMR server for nodal authorities to track and isolate the COVID-19 infected individuals and also managing large population simultaneously. The application works in alliance with Indian government's digital India initiative. This is also the first use of artificial intelligence (AI) based algorithms for interpretation of test results at such a mass scale and reduces the errors caused by manual interpretations. The app is also provided with a provision to report oneself as self-reported positive in case of negative results based on the AI-based image analysis but the person is symptomatic. The present measure has limitation for usage in rural area where the users have no or limited access for the smartphones.

Overall, our observations and experience suggest that time has come for India to embrace the self-test's for other diseases too. It opens the opportunity for the development of RAT for other opportunistic diseases such as Dengue fever, Diphtheria, Hepatitis, Malaria to mention a few. These tests can be linked to various disease control programs conducted by the Govt of India with or without support from world health organization (WHO). The way forward to control spread of any disease is its timely diagnosis. If self-test kits are promoted and the public in general is made aware of how to conduct them, it will be a paradigm shift in a country like India where population density itself makes healthcare inaccessible and unaffordable.

## Conclusion:

The results of this study points towards the idea that self-testing kit is an effective diagnostic tool especially for large or underserved populations. The overall response in terms of ease of sample collection, safety, efficacy and testing is comparable across user members of different socioeconomic status, gender and age groups. Users have reported that the kit is very user friendly and can be used very effectively by the common public and does not demand any specific training. Quoting from popular culture, it seems that self-testing kits seems to be the idea whose time has come.

## Abbreviations:

RT-PCR: Reverse transcriptase polymerase chain reaction; NGS: Next-generation sequencing; RAT: rapid antigen test; ICMR: Indian council of medical research; ANOVA: Analysis of variance.

## Declaration:

## Ethics approval and consent to participate:

The study has been done on the ICMR approved self-test kit available in the market. However, written informed consent has been obtained from the participants.

## Availability of data and materials:

All the data has been reported in the manuscript. However in case some data is needed that will be made available on request to corresponding author.

## Competing interests:

All authors declare no financial or any conflict of interest.

## Funding:

The study was funded Mylab Discovery Solutions Pvt. Ltd. Pune.

## Authors' Contributions:

DSC, VG SG and SP conceived the study. VS and SP designed the questionnaire. DSC and VG conducted the survey under the supervision of SP. KB contributed in the design of Mylab CoviSelf mobile application. DSC analyzed the data and prepared the first draft of the manuscript. DSC, SP and SG contributed in the manuscript revision. SP supervised the study.

## Figures and Tables

**Table 1:** Table illustrating the observer’s user related feedback on use of CoviSelf™ COVID-19 Ag Test kit.

**Observations**	**Population (%)**
User opened the sterile swab pouch before testing	97
User handled sterile swab carefully and effectively collected the sample	93
User correctly dipped the swab into the buffer	94
Final extract with mucus	17
User fitted the nozzle properly after the extraction	93
User handled the test cassettes properly	93
User dropped the excess extraction buffer on the cassette	8
User correctly interpreted the result	92
User interpreted the result within 15-20 minutes of the test	99
User followed the proper disposal process after test	92

**Table 2:** overall satisfaction scores offered by participants from different socioeconomic status to the eight study parameters

**Parameters**	**DWW_TH**	**OS_BN**	**OS_CH**	**IS_UN**	**FW_IT**	**FW_HD**	**FW_CH**	**HH_KS**
Patients Safety	4.7	5	4.9	5	5	5	4.7	4.7
Effectiveness	4.5	5	4.7	4.5	4.8	5	5	4.7
Ease of Sample Collection	4.5	5	4.8	5	5	4.5	5	4.3
Ease of Testing Process	4.5	5	5	5	5	5	5	4.7
Ease of Result Interpretation	4.5	5	4.7	4.8	3.8	4.5	5	5
Ease of Understating the User Manual and Labels	4.1	4.9	4.5	4.3	4	5	5	5
Ease of Understating the Consequences if steps are not performed correctly	4.2	4.6	4.7	4.5	4	5	4.3	5
Overall satisfaction with the Ag Test Kit	4.6	4.9	4.9	4.8	4	5	4.7	5
DWW_TH; Daily wage workers (Tathawade), OS_BN; Office staff (Baner), OS_CH; Office staff (Chinchwad), IS_UN; industry staff (Undri), FW_IT; Factory worker (ITC), FW_HD; Factory worker (Hadapsar), FW_CH; Factory worker (Chinchwad), HH_KS; Household members (Karishma society)

**Table 3:** Overall satisfaction scores offered by percent participants to the eight study parameters

**Parameters**			**Satisfaction scores offered by percent population**		
	**Score_01**	**Score_02**	**Score_03**	**Score_04**	**Score_05**
Patients Safety	-	-	4.7	19.3	76
Effectiveness	-	1.3	5.3	27.3	66
Ease of Sample Collection	0.7	2	8.7	12.7	76
Ease of Testing Process	0.7	3.3	4	14	78
Ease of Result Interpretation	2.7	2	4.7	20.7	70
Ease of Understating the User Manual and Labels	4	4	10	24	58
Ease of Understating the Consequences if steps are not performed correctly	3.3	1.3	9.3	29.3	56.7
Overall satisfaction with the Ag Test Kit	0.7	0.7	8	15.3	75.3
